# Petersen's Space Internal Hernia after Laparoscopic One Anastomosis (Mini) Gastric Bypass

**DOI:** 10.1155/2018/9576120

**Published:** 2018-04-01

**Authors:** Mohammad Kermansaravi, Mohammad Kazazi, Abdolreza Pazouki

**Affiliations:** ^1^Minimally Invasive Surgery Research Center, Iran University of Medical Sciences, Tehran, Iran; ^2^Center of Excellence of International Federation for Surgery of Obesity, Hazrat-e Rasool Hospital, Tehran, Iran; ^3^Shahid Lavasani Hospital, Social Security Organization, Tehran, Iran

## Abstract

**Background:**

One anastomosis gastric bypass (OAGB) is now considered as an appropriate alternative for Roux-en-Y gastric bypass (RYGB) with some advantages such as absence of risk for internal hernia (IH). But, is really the risk of IH equal zero after OAGB? *Case Summary*. A 37-year-old male was admitted due to severe abdominal crampy pain, nausea, vomiting, and obstipation. He had chronic and intermittent abdominal pain from 2 years after OAGB. With high suspicion of complete obstruction, the exploratory laparoscopy was performed. Intraoperative findings showed incarcerated bowel hernia from Petersen's defect. The incarcerated bowel was reduced, and the defect was repaired. The patient was discharged 2 days after operation.

**Conclusion:**

The incidence of IH after OAGB is rare but not zero. In any suspicious signs and symptoms for IH, the early exploratory laparoscopy is mandatory to diagnose and treat.

## 1. Introduction

One anastomosis gastric bypass (OAGB) or mini-gastric bypass is now accepted around the world as an alternative method for Roux-en-Y gastric bypass (RYGB), the gold standard method of bariatric surgery, with comparable efficacy and safety and some advantages to RYGB [1, 2]. Absence of internal hernia is considered as one of the advantages of OAGB in long-term follow-ups [1–3]. Petersen's space is the space between the afferent loop mesentery of gastrojejunostomy and the lower part of transverse colon mesentery that is created after gastrojejunostomy in some procedures such as RYGB and OAGB [3, 4]. Till now, only two Petersen's space hernia (PH) after OAGB are reported [3, 5] showing that PH occurrence is rare after OAGB; however, it is not impossible.

## 2. Case Presentation

**Figure 1:**
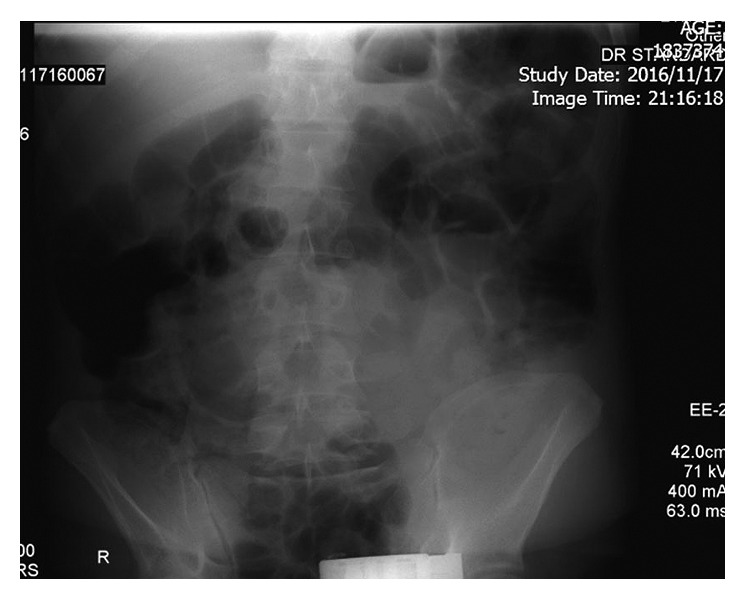
Plain abdominal X-ray during abdominal pain attack.

A 37-year-old male, with body mass index (BMI) of 27 kg/m^2^, was admitted in emergency department with severe crampy abdominal pain, nausea, vomiting, and obstipation from 2 days before admission. He had a history of OAGB (with concomitant Braun jejunojejunostomy 30 cm below the gastrojejunostomy) 3 years ago. Twenty-seven months after OAGB, he had complaints of crampy and intermittent left upper quadrant (LUQ) abdominal pain episodes, which led to two times hospital admissions before. Clinical and paraclinical evaluations such as upper endoscopy, abdominal ultrasonography, abdominal radiographies, and computed tomography (CT) scan with IV/PO contrast had reported normal findings. One of his abdominal plain X-rays is shown in Figure 1. The patients had a BMI loss of 20.9 kg/m^2^ during 3 years after surgery.

During this admission, on physical examination, he had normal temperature, blood pressure, and respiratory rate; however, he had tachycardia (HR: 110/min). The abdomen was distended without any tenderness and guarding.

## 3. Management

**Figure 2:**
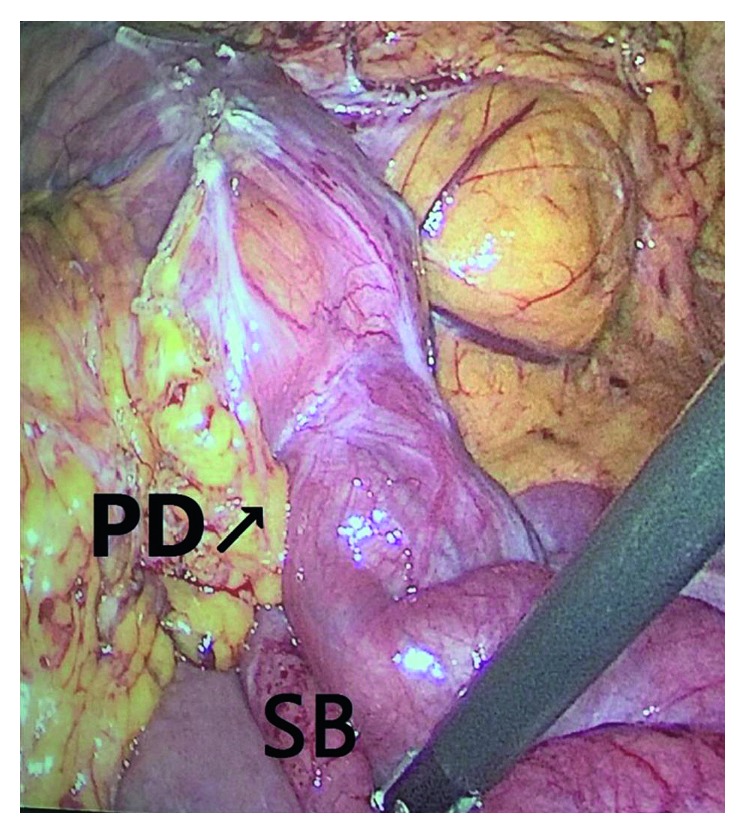
Intraoperative view of IH that shows herniated small bowel (SB) through Petersen's defect (PD).

**Figure 3:**
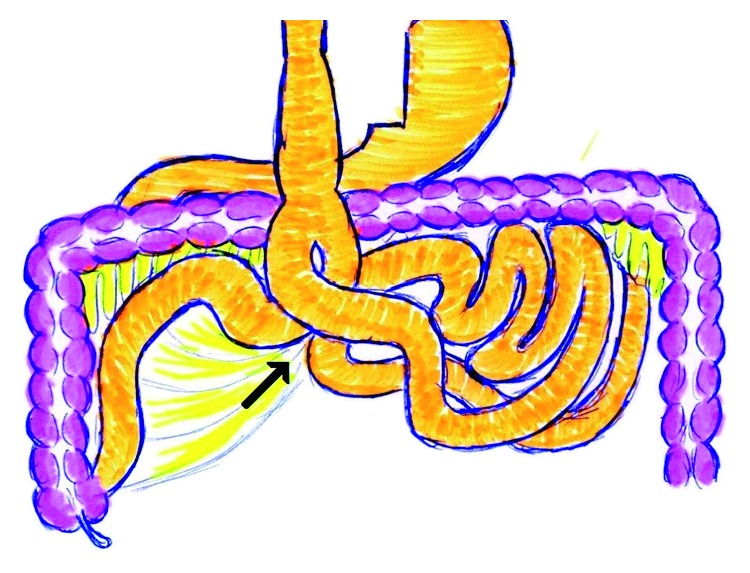
Schematic picture in this case shows the herniation of whole small bowel from Petersen's space (arrow).

Due to high suspicion of complete obstruction, after initial resuscitation, the patient underwent exploratory laparoscopy. During laparoscopy, most of the small bowel had been passed through the Petersen's space from right to left and was incarcerated with few patchy lesions in some sites of the small bowel without any sign of severe ischemia and gangrene (Figures 2 and 3). The incarcerated bowel was reduced, and the defect was sewn with Prolene 2-0. Also Braun's jejunojejunal defect was closed in order to prevent the subsequent IH. He was discharged 2 days after operation and had no problem till 6 months of follow-up.

## 4. Discussion

Internal hernia (IH) occurs after some malabsorptive surgical procedures such as RYGB [3], with an incidence between 0.9% and 11.7%, if the defect was not closed after RYGB [6–8]. Defect closure can reduce the incidence of IH but cannot eliminate it [7, 9]. Aghajani et al. in a study with 60 months of follow-up on 2443 patients who underwent LRYGB showed that defect closure leads to reduction of IH incidence from 11.7% to 2.5% and 4.09-fold decrease of relative risk for IH [7].

Due to longer pouch creation in OAGB in comparison to RYGB and therefore larger Petersen's space, the risk of incarceration and strangulation is very low [2], as in two past case reports, there was no evidence of bowel ischemia [3, 5]. Although in this case that the most of the bowel length was herniated, it is possible to have incarceration and ischemia. On the other hand, the rare incidence of IH after OAGB and the fact that defect closure addition to operating time elongation may lead to some complications such as mesenteric bleeding and hematoma, kinking and rotation of anastomosis, and also adhesion formation [10]; it could not be recommended to routine closure of Petersen's space during OAGB; however, in presence of more possible reports over time, the traditional approach needs to be changed.

The most important lesson is that chronic and intermittent abdominal pain, especially in LUQ, and also nausea especially after meal, can be a presentation of IH after RYGB and OAGB [3–5]. Up to 20% of IH could have normal findings in CT scan and small bowel series [4], so in high suspicious conditions for IH, as the same as RYGB, prompt explorative laparoscopy is necessary for the achievement of definite diagnosis and appropriate treatment to prevent possible serious complications [4, 10].
